# Comparative study between biopsy and brushing sampling methods for detection of human papillomavirus in oral and oropharyngeal cavity lesions^☆^^☆☆^

**DOI:** 10.1016/j.bjorl.2015.08.007

**Published:** 2015-09-08

**Authors:** Marise da Penha Costa Marques, Ivo Bussoloti Filho, Lia Mara Rossi, Maria Antonieta Andreoli, Natália Oliveira Cruz

**Affiliations:** a Faculdade de Ciências Médicas, Santa Casa de São Paulo (FCMSCSP), São Paulo, SP, Brazil; b Hospital Universitário Clementino Fraga Filho, Universidade Federal do Rio de Janeiro (UFRJ), Rio de Janeiro, RJ, Brazil; c Department of Otorhinolaryngology, Irmandade da Santa Casa de Misericórdia de São Paulo, São Paulo, SP, Brazil; d Faculdade de Medicina de Jundiaí, Jundiaí, SP, Brazil; e Instituto Nacional de Ciência e Tecnologia das Doenças Associadas ao Papilomavírus, São Paulo, SP, Brazil

**Keywords:** Polymerase chain reaction, Human papillomavirus DNA tests, Mouth, Oropharynx, Reação de polimerase em cadeia, Testes de DNA para papilomavírus humano, Boca, Orofaringe

## Abstract

**Introduction:**

Many epidemiological studies have suggested that human papillomavirus (HPV), especially type 16, is involved in the genesis of squamous cell carcinoma of the oral cavity and oropharynx, especially in young, non-smoking patients; thus, its detection in lesions in this region is important.

**Objective:**

To clarify the capacity of the brushing sampling method to detect the presence of HPV in oral or oropharyngeal lesions through polymerase chain reaction (PCR) testing, and to compare the results with those obtained by biopsy.

**Methods:**

Prospective study of adult patients with oral or oropharyngeal lesions assessed by PCR, comparing biopsy specimens with samples obtained by the brushing method. The study was approved by the Research Ethics Committee of the institution.

**Results:**

A total of 35 sample pairs were analyzed, but 45.7% of the brushing samples were inadequate (16/35) and, thus, only 19 pairs could be compared. There was agreement of results in 94.7% (18/19) of the pairs, with HPV identified in 16 of them. HPV DNA was detected in 8.6% (3/35) of biopsy and 5.7% (2/35) of brushing samples.

**Conclusion:**

There was no statistically significant difference between the two methods, but the brushing sampling method showed a higher number of inadequate samples, suggesting that it is an unreliable method for surveillance.

## Resumo

**Introdução:**

Muitos estudos epidemiológicos indicam a participação do papilomavírus humano, especialmente o tipo 16, na carcinogênese dos tumores espinocelulares das cavidade oral e orofaríngea, principalmente em jovens e não fumantes, sendo portanto importante sua detecção nas lesões desta região.

**Objetivo:**

Elucidar a habilidade do escovado em detectar o papilomavírus humano, pela reação em cadeia da polimerase, nas lesões orais e orofaríngeas, comparando os resultados com os obtidos por biópsia.

**Método:**

Estudo prospectivo de pacientes com lesões orais e orofaríngeas, pela reação em cadeia da polimerase, no qual foram pareados os resultados de amostras obtidas por escovado e por biópsia. A pesquisa foi aprovada pelo Comitê de Ética em Pesquisa da instituição.

**Resultado:**

Foram analisados 35 pares de amostras, porém estavam inapropriadas para análise 45,7% (16/35) das amostras obtidas por escovado, e portanto, somente 19 pares puderam ser comparados. Em 94,7% dos pares houve concordância dos resultados, sendo encontrado o papilomavírus humano – 16 em um destes pares. O ácido desoxirribonucleico do papilomavírus humano foi detectado em 8,6% (3/35) das biópsias e em 5,7% (2/35) dos escovados.

**Conclusão:**

Não houve diferença estatística entre os métodos, mas como houve um grande número de amostras obtidas por escovado inapropriadas, este parece não ser confiável para o rastreamento.

## Introduction

Squamous cell carcinoma (SCC) comprises more than 80% of the mouth and oropharynx carcinomas and its incidence in the head and neck has been increasing over the last thirty years, especially in non-smokers and patients younger than 45 years of age.^1^, ^2^, ^3^, ^4^ Syrjänen et al. (1983) were the first to suggest that human papillomavirus (HPV) could also be involved in this carcinogenesis as it is in cervical carcinoma; since then, many studies have been performed to establish the prevalence of HPV in the mouth and oropharynx, both in patients with and without lesions.^1^, ^2^, ^5^, ^6^

For these reasons, it appears important to establish an affordable and reliable surveillance method for clinical or subclinical infection with high-risk HPV in oral and oropharyngeal mucosa for head and neck SCC prevention. HPV detection methods in SCC of the mouth and oropharynx show broad variations in sensitivity and specificity, with prevalence ranging between 0% and 78%; thus, it is very important to choose a method that has high sensitivity and specificity for HPV detection.

Currently, the most often used method is reverse hybridization with degenerate primers labeled with biotin found in commercial kits, which allows the genotyping of most types of high and low-risk HPV. There are many factors that can affect viral detection, such as lesion location, presence or absence of keratinization, type of sample collected, and collection procedure (how the sample was collected, preserved, and extracted), in addition to the methods used in detection.^2^, ^7^, ^8^, ^9^

Biopsy remains the preferred method for obtaining otopharyngeal lesion material, since, in addition to providing a more detailed morphological study, the biopsy sample allows the recovery of basal layer cells, where the HPV could be found in its latent form.^3^, ^10^ However, it is a relatively expensive method, as it requires the presence of a physician and surgical material, which are not always available in the service unit.

This study aimed to compare, through polymerase chain reaction (PCR) and linear array hybridization, HPV presence in material collected by the brushing sampling method and biopsy of mouth and oropharynx lesions, testing the viability of the brushing sampling for collection of material from mouth and oropharynx lesions.

## Methods

This was a prospective cross-sectional study of 35 volunteers with oral or oropharyngeal lesions with indication for biopsy, consecutively treated at the otorhinolaryngology clinic of a general hospital, from April of 2012 to December of 2012, who met the following inclusion criteria: individuals aged >21 years of age, with white or red, vegetating, infiltrating, and/or ulcerated lesions in the oral or oropharyngeal cavity lasting more than 15 days. The exclusion criteria were clinical contraindication to the surgical procedure and antiviral use. HPV screening results were compared in the material obtained by brushing sampling method and biopsy of the same lesion.

The project was approved by the Human Research Ethics Committee, registered under No. 192/09. Before being submitted to material collection, the selected patients signed the informed consent and answered a questionnaire on epidemiological data that included age, gender, tobacco and alcohol consumption, number of sexual partners during their lifetime and in the last six months, type of partner, and the duration and location of the lesion.

Collection of the biological samples was performed by the same professional in a surgical environment, using aseptic techniques and locoregional infiltrative anesthesia with 1% lidocaine. Material collection was first performed using the brushing sampling method, by rubbing a Cytobrush Plus™ brush over the lesion using three forward–backward movements, followed by the biopsy performed with a scalpel blade, avoiding areas of necrosis and, whenever possible, including tissue adjacent to the lesion.

The brush with the material was stored in a cryovial containing 0.9% aqueous saline solution, which was immediately frozen in liquid nitrogen at −170 °C. Subsequently, the biopsy was performed and the material was divided into three fragments: the first was placed in a 10% formaldehyde buffered aqueous solution for anatomopathological analysis, the second was used in this research, and the third was stored at the Biobank. The fragments were placed in separate and dry cryotubes and immediately frozen in the same container. All samples were transported to the freezer together, remaining frozen at −80 °C until they were processed by the Molecular Biology Laboratory.

Samples were processed according to existing biosecurity standards. The in-house method was used for DNA extraction, in which the sample was digested with proteinase K, followed by purification with phenol/chloroform/isoamyl alcohol (25:24:1, Invitrogen) and quantified in a Nanodrop1000 spectrophotometer (Thermo Scientific).

The quality of the obtained DNA was verified by performing PCR of human β-globin with the PCO3/PCO4 primer with 110-bp amplicon (Saiki et al.). Both positive and negative samples for human β-globin were genotyped using a Linear Array Hybridization kit (Roche Diagnostics), which allows the identification of 37 types of HPV of high and low risk through linear reverse hybridization.

The statistical analysis of this study was descriptive, with the help of measures of location, and the results are shown in Table 1. The “*z*” test was used for quantitative variables. The null hypothesis was no significant difference between the two proportions, with a significance level of 0.05. The XLSTAT 2013.4.02 program was used to test the two proportions, with a right-tailed one-sided, 95% confidence interval for the difference between proportions.

**Table 1:** Characteristics of the samples and results obtained by PCR of the β-globin gene, viral DNA detection, genotyping by linear array hybridization of the material obtained by biopsy, and brushing sampling methods.

Case	Characteristics of lesions	Brushing sample		Tissue sample	
		β-Globin	DNA HPV	β-Globin	DNA HPV
1	Tongue SCC	Yes	No	Yes	No
2	Palatine tonsil SCC	Yes	No	Yes	No
3	Oral mucosa SCC	Yes	No	Yes	No
4	Palatine tonsil SCC	Yes	HPV-52	Yes	No
5	Ulcerated inflammatory process of palatine tonsil	Yes	No	Yes	No
6	Papillomatosis of oral cheek mucosa	Yes	No	Yes	No
7	Base of the tongue SCC	Yes	No	Yes	No
8	Ulcerated inflammatory process of the mouth floor	No	No	Yes	No
9	Hodgkin's lymphoma of base of the tongue	No	No	Yes	HPV-6
10	Palatine tonsil SCC	No	No	Yes	No
11	Soft palate papillomatosis	No	No	Yes	No
12	Soft palate papillomatosis	No	No	Yes	No
13	Mouth floor SCC	No	No	Yes	No
14	Lower-lip SCC	No	No	Yes	No
15	SCC of the base of the tongue	No	No	Yes	No
16	Ulcerated inflammatory process of the oral cheek mucosa	No	No	Yes	No
17	Tongue SCC	Yes	No	Yes	No
18	Soft palate papillomatosis	No	No	Yes	No
19	Palatine tonsil papillomatosis	No	No	Yes	No
20	SCC of the base of the tongue	No	No	Yes	No
21	Oropharyngeal squamous papilloma	No	No	Yes	HPV-11
22	Non-Hodgkin lymphoma of palatine tonsil	Yes	No	Yes	No
23	Soft palate SCC	Yes	No	Yes	No
24	Tongue SCC	Yes	No	Yes	No
25	Palatine tonsil SCC	No	No	Yes	No
26	Palatine tonsil SCC	Yes	No	Yes	No
27	Palatine tonsil SCC	Yes	HPV-16	Yes	HPV-16
28	SCC of the base of the tongue	Yes	No	Yes	No
29	Tongue squamous papilloma	No	No	Yes	No
30	Fibroepithelial polyp of the soft palate	Yes	No	Yes	No
31	Squamous papilloma of the anterior pillar	Yes	No	Yes	No
32	SCC of the uvula	Yes	No	Yes	No
33	Soft palate SCC	Yes	No	Yes	No
34	Tongue SCC	Yes	No	Yes	No
35	Soft palate SCC	No	No	Yes	No

HPV, human papillomavirus; DNA, deoxyribonucleic acid; SCC, squamous cell carcinoma.

The literature review was conducted online, using the databases of the US National Library of Medicine of the National Institutes of Health (PubMed) and the *Biblioteca Virtual em Saúde* (LILACS), using the following subject descriptors: polymerase chain reaction, human papillomavirus, oral mucosa, oropharyngeal mucosa, oropharynx, detection, brushing, and biopsy.

## Results

A total of 35 individuals were evaluated, 26 men and nine women, with an approximate male to female ratio of 3:1. Age ranged from 37 to 77 years, with a mean of 54 years. All individuals declared they were heterosexuals. Regarding social habits, 11 (31.4%) had never consumed alcohol regularly, 18 (51.4%) were not current users, and six (17.1%) still consumed; four (11.4%) had never smoked, 11 (31.4%) were ex-smokers, and 20 (57.1%) were current smokers.

SCC was identified in 21 (60%) of 35 lesions (21/35); of these, 15 (71.4%) were moderately differentiated (15/21), three (14.3%) were well-differentiated (3/21), and three (14.3%) were poorly differentiated (3/21). Of the remaining lesions (14/35), five (35.7%) received an anatomopathological diagnosis of papillomatosis, three (21.4%) of squamous cell papilloma; three (21.4%) of ulcerated chronic inflammatory processes, two (14.3%) of lymphoma, and one (7.2%) of fibroepithelial polyp.

As for lesion location, ten (28.6%) were located on the tongue, of which six were at the base of the tongue; nine (25.7%) in the palatine tonsil; seven (20%) in the soft palate; three (8.6%) in the cheek mucosa; two on the mouth floor (5.7%), and one each (2.9%) in the following locations: anterior pillar, uvula, lower lip, and oropharynx mucosa.

All samples obtained through biopsy were 100% positive for β-globin; in contrast, in samples obtained by the brushing sampling method, positivity was 54.3% (19/35). Nineteen pairs were compared; in 18, there was agreement concerning the presence or absence of HPV DNA. Of the biopsy samples, three lesions were positive for HPV DNA: type HPV-16 in a patient also submitted to the brushing sampling method, type HPV-6 in a patient with base of the tongue lymphoma and HPV-11 in an oropharynx papilloma. For brushing sampling cases, HPV DNA was isolated from two cases of moderately differentiated palatine tonsil SCC (Table 1).

### Statistical analysis

Of the 35 samples obtained by the brushing sampling method, only 19 were analyzed, which were positive for the β-globin reaction, and of these, two were positive for HPV (2/19), with a proportion of 0.105263. As for the tissue samples obtained by biopsy, 100% were positive for β-globin, with three being positive for HPV, with a proportion of 0.085714. Only a pair of positive samples showed agreement with the genotyping of the correspondent sample obtained by the brushing method. The XLSTAT 2013.4.02 program was used to test the two proportions. The *z*-test for two proportions was right-tailed and one-sided, with a 95% confidence interval. The difference between the proportions was 0.020 and the *z* (observed value) was 0.230, with *z* (critical value) of 1.645 and *p*-value (one-sided) of 0.409; showing that there was no statistically significant difference between the proportions of HPV in both types of samples.

## Discussion

Head and neck tumors have a global incidence of 460,000 new cases per year, leading to 228,000 deaths estimated in 2014, according to the National Cancer Institute of Brazil. HPV-16 is responsible for 25% of all squamous cell carcinomas of the head and neck, and it is present in 45–90% of all cases of oropharynx tumors and approximately 24% of larynx and oral cavity tumors.^3^

For this study, the number of cases was established based on the literature, where the prevalence of HPV in mouth and pharynx lesions is approximately 36% (*p* = 0.36), with an estimated error of 3% to define the sample size required for the analysis.^11^, ^12^, ^13^, ^14^, ^15^, ^16^, ^17^

In Brazil, in individuals without lesions and with small samples up to 100 individuals, the prevalence of HPV ranged from 0% to 12%,^18^, ^19^, ^20^, ^21^ whereas in other countries, the prevalence ranged from 0%^22^ to 97%,^23^ suggesting that the prevalence of oral and oropharyngeal infection by HPV in the Brazilian population is low; however, a population screening study is necessary to better understand the real prevalence of HPV in the oropharyngeal cavity of the Brazilian population. In this study, the high-risk HPV types, HPV-16 and HPV-52, and the low-risk types, HPV-6 and HPV-11, were identified, with an overall prevalence of 11.4%. The prevalence of HPV in oral and oropharyngeal lesions is very wide-ranging, varying from 0%^24^ to 77.8%^9^ and in national studies ranging from 0%^7^ to 75%^19^; it was not possible to reliably compare the results, as the studies had very different methodologies.

The prevalence of HPV is higher in patients who had other sexually transmitted diseases,^25^ with a large number of oral sex partners a predictive factor for oral HPV detection.^23^ However, contrary to what has been expounded, study subjects who had more than twenty sexual partners did not have HPV DNA detected in their lesions.

To date, there is no consensus on the best sample collection technique for patients with oral and oropharynx lesions; the development new studies are required, and thus it is important to compare the methods.

When the brushing and biopsy sampling methods were compared in this study in samples with mouth or oropharynx SCC, there was no statistical difference between the methods, with 94.7% (18/19) agreement in pairs of samples. Termine et al., in a similar study, but not in oropharyngeal lesions, observed that the frequency of detection through brushing and biopsy sampling methods also showed no statistically significant difference, but the biopsy method showed to be more accurate in high-risk HPV detection.^26^

Lawton et al. assessed three sampling methods and concluded that mouth-rinsing, when used alone is the best method, but positivity can be increased when combined with other methods for material collection.^27^ Jarboe et al. evaluated the effectiveness of the hybrid capture in two oral brushing and oral rinsing samples, finding greater detection in brushed samples than in those from mouth-rinsing.^28^ Read et al. compared three methods, and the mouth-rinsing method showed higher detection sensitivity, especially in those individuals who had brushed their teeth before rinsing.^23^

Even though the brushing sampling method was performed according to standardized procedures and following previously established protocols, 45.7% of the samples (16/35) were inadequate for HPV screening, suggesting low sensitivity of the collection method, or incapacity of the brush, intended for use in gynecological collection, to obtain material from the mouth and oropharynx lesions, or because the medium in which samples were placed was not able to preserve them. Perhaps the choice of fixation and preservation solutions to be used with the brushing sampling method, such as PreservCyt™ solution,^2^ Digene™ solution,^29^ and phosphate-buffered saline (PBS)^2^, ^15^, ^26^, ^29^, ^30^, ^31^ may result in better samples for cytological analysis than those placed in saline solution, as that used in this research. The comparison between the fixation solutions of the mouth and oropharynx materials collected through the brushing sampling method could clarify this doubt.

These results confirm the involvement of HPV in oral and oropharyngeal lesions, but further studies are needed to detail the collection method, with the investment in specific kits that can detect scarce cells, which will allow better identification of HPV in these HPV-related oral and oropharyngeal lesions, resulting in improved prevention and treatment.

## Conclusions

There was no statistical difference between the brushing and biopsy methods for detection of viral DNA in oral and oropharyngeal lesions, but the large number of inadequate samples obtained by brushing suggests this method is inefficient when obtaining samples from this type of lesion.

## Funding

This study was funded by 10.13039/501100001807FAPESP.

## Conflicts of interest

The authors declare no conflicts of interest.
